# Advertising by Physicians

**Published:** 1877-09

**Authors:** 


### EDITORIAL.

### Advertising by Physicians.

All respectable Medical Associations condemn the practice of advertising as only intended to deceive, cheat and rob the uneducated public--the poor mechanic, the hopeless consumptive, the sufferer from cancer or other incurable malady. Medical advertising is for the express purpose of deception, and of course no true, honest physician can deceive. To desire to do so makes him a knave and a quack. But there is a prevalent desire to advertise, not to misrepresent, perhaps, but to be fairly and truthfully placed before the public, to have general attention directed to real merits by a short-cut route. Patient waiting is too long a journey, and in these days of competition in business, cannot be tolerated, so that we have, in spite of the professional sentiment against it, a manifest desire to advertise It is not the straight, manly, fair, honorable advertisement of a business firm having capital and honest purpose to fulfill the bill ; it is a miserable underhanded *advertising dodge,* designed to avoid the responsibility to not assume it. It is covered by some, so-called, charitable, free to the poor, dispensary, infirmary, sanitarium or other bogus institution, with a long list of specialists squarely advertised as experts of experience in the various departments of special practice. All this advertising we most thoroughly despise. We have no hesitancy in saying that this covered, underhanded, concealed plan of procedure is more demoralizing to the profession, more injurious to good morals, good manners and good practice, than would be a complete, business-like, manly and reasonably truthful advertisement in the *usual way.*

Considering the situation we are in favor of allowing all physicians who desire, and are spoiling for want of it, to advertise in the regular way, furnish their own copy in their own words, and not any more invite newspaper reporters to be present. The humiliation of the present condition cannot be borne. We prefer advertising to it, and we would regard doctors employing the city cryer to announce their names, office location and hours, with details of remarkable cures, as more professional, honest and respectable, than this covert, unreal, dishonest and dead head advertising. We find fault with the quacks because they advertise dishonestly, and offer superior practice, but the profession in considerable degree, carry on the same business under cover, and avoid paying the printer.

